# Development features and study characteristics of mobile health apps in the management of chronic conditions: a systematic review of randomised trials

**DOI:** 10.1038/s41746-021-00517-1

**Published:** 2021-10-05

**Authors:** Maria Cucciniello, Francesco Petracca, Oriana Ciani, Rosanna Tarricone

**Affiliations:** 1 University of Edinburgh Business School - Centre for Service Excellence (CenSE), Scotland, UK; 2 Centre for Research in Health and Social Care Management (CERGAS), Government, Health and Non Profit Division, SDA Bocconi, Via Sarfatti 10, 20136 Milan, Italy; 3 Institute of Health Research, University of Exeter Medical School, South Cloisters, St. Luke’s Campus, Heavitree Road, EX1 2LU Exeter, UK; 4 Department of Social and Political Sciences, Bocconi University, Via Roentgen 1, 20136 Milan, Italy

**Keywords:** Health policy, Health services

## Abstract

COVID-19 pandemic challenges have accelerated the reliance on digital health fuelling the expanded incorporation of mobile apps into healthcare services, particularly for the management of long-term conditions such as chronic diseases (CDs). However, the impact of health apps on outcomes for CD remains unclear, potentially owing to both the poor adoption of formal development standards in the design process and the methodological quality of studies. A systematic search of randomised trials was performed on Medline, ScienceDirect, the Cochrane Library and Scopus to provide a comprehensive outlook and review the impact of health apps on CD. We identified 69 studies on diabetes (*n* = 29), cardiovascular diseases (*n* = 13), chronic respiratory diseases (*n* = 13), cancer (*n* = 10) or their combinations (*n* = 4). The apps rarely adopted developmental factors in the design stage, with only around one-third of studies reporting user or healthcare professional engagement. Apps differed significantly in content, with a median of eight behaviour change techniques adopted, most frequently pertaining to the ‘Feedback and monitoring’ (91%) and ‘Shaping knowledge’ (72%) categories. As for the study methodologies, all studies adopted a traditional randomised control trial (RCT) design, with relatively short follow-ups and limited sample sizes. Findings were not significant for the majority of studies across all CD, with most RCTs revealing a high risk of bias. To support the adoption of apps for CD management, this review reinforces the need for more robust development and appropriate study characteristics to sustain evidence generation and elucidate whether study results reflect the true benefits of apps or a biased estimate due to unsuitable designs.

## Introduction

Smartphone users in the world have steadily increased, surpassing the 3.5 billion mark in 2020 with a further expected growth of several hundred million in the coming years^1^, fuelling the expanding interest in mHealth apps^2^. The coronavirus disease 2019 (COVID-19) pandemic has contributed to further accelerate the reliance on digital health^3–5^.

Therefore, apps are being increasingly incorporated into healthcare (HC) services owing to their portability, instantaneous access and direct communication, inspiring new models of remote HC delivery and cost-effective solutions for chronic diseases, whose long-term nature and need for continuous monitoring can be positively impacted^2,6,7^. mHealth apps may be particularly effective in self-management, one of the components of the eHealth Enhanced Chronic Care Model^8^, intended as the ‘individual’s ability to manage the symptoms, treatment, physical and psychosocial consequences and lifestyle changes inherent in living with a chronic disease’^9^. Apps could as well improve patient empowerment, the process of gaining knowledge of one’s health and ability and motivation to influence it^10,11^.

Despite the ever-growing interest and the increasing number of apps on common platforms, their impact on outcomes for chronic diseases remains unclear. Several systematic reviews and meta-analyses reported mixed impact of apps on clinical outcomes, self-management and behaviour change^12–21^, questioning the ability of science to keep up with the continuous technological advances^22^.

The inconclusive evidence base of apps could be attributed both to the poor adoption of formal development standards^23,24^, notwithstanding the reported benefits of adopting participatory approaches and behaviour change theories^25,26^, and to the methodological quality of study designs^27^.

Previous reviews had summarised the impact of apps on health outcomes across different conditions, concluding that high-quality research is needed to transform the promise of mHealth technology into improved HC delivery and outcomes^7,28^. More recently, Iribarren et al. comprehensively reviewed app-delivered behaviour change interventions targeting health outcomes and thoroughly analysed the corresponding app features^29^. However, no study on chronic diseases has comprehensively looked at both the developmental features of apps, namely the strategies and considerations adopted throughout the development stage of mobile apps, thereby including the behavioural change features adopted, and the characteristics of the evaluation study in determining the effect on a wide array of domains.

We, therefore, conducted a systematic review of randomised studies on smartphone apps for the management of high prevalence diseases (diabetes, cardiovascular diseases, chronic respiratory diseases and cancer) to fill this gap.

## Results

### Study selection

Following the removal of duplicates, the addition of 16 publications cited in reference lists, and title and abstract screening, 175 records were selected for full-text examination. The Cohen’s kappa coefficient expressing the inter-rater agreement was 0.84 during the abstract screening.

**Fig. 1:**
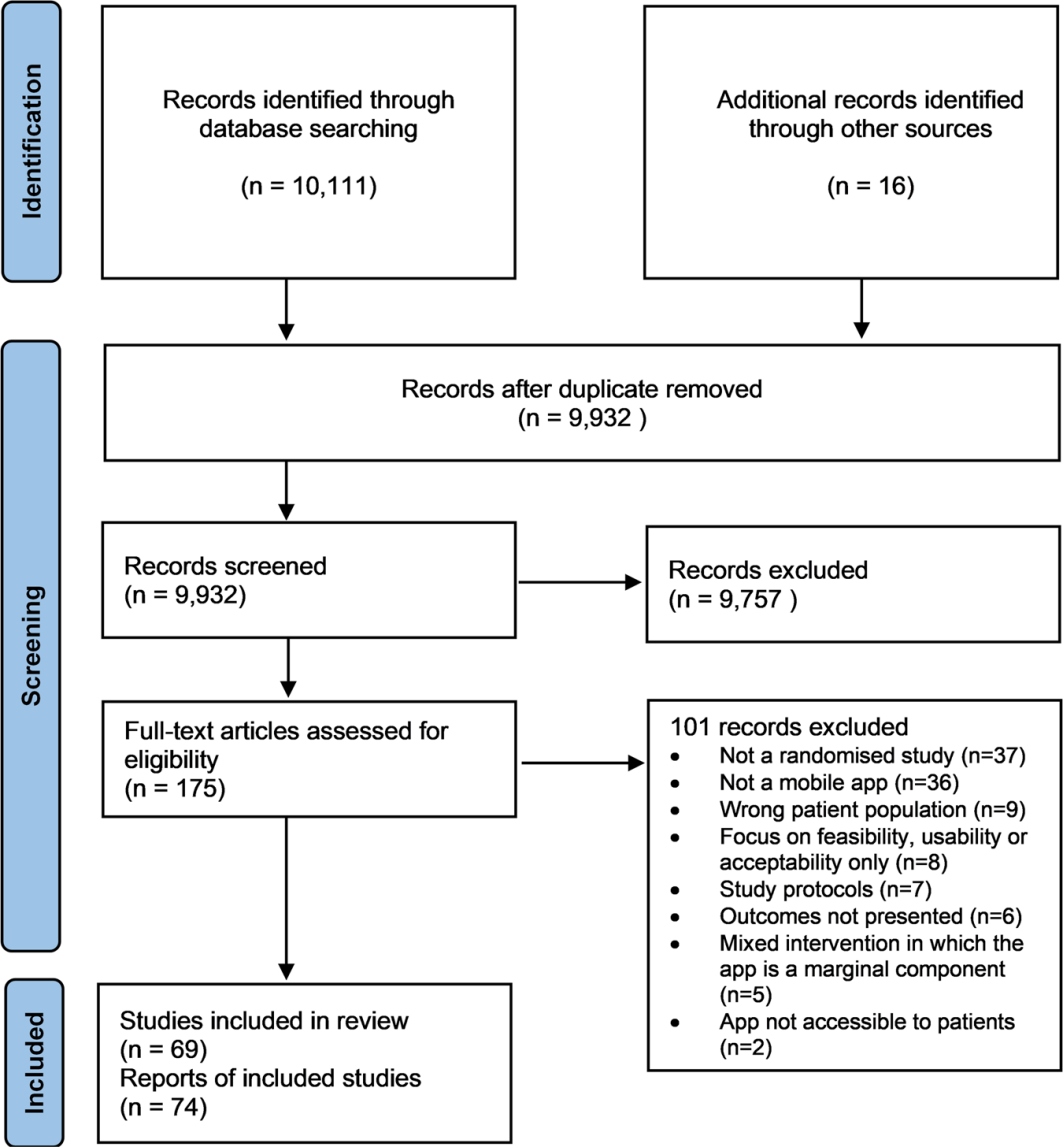
Study selection flow diagram. Modified Preferred Reporting Items for Systematic Reviews and Meta-Analyses (PRISMA) flow diagram of the article selection process.

Overall, 74 papers based on 69 different trials were included in the review. The Preferred Reporting Items for Systematic Reviews and Meta-Analyses (PRISMA) flowchart of the study selection process is presented as Fig. 1, including the reported reasons for exclusion.

### Characteristics of included studies and participants

The studies were published between 2008 and 2019 in 24 countries, with the United States of America (*n* = 13)^30–43^ and China (*n* = 7)^44–50^ ranked first and second, respectively. Only one study covered multiple countries, involving diabetes clinics in Italy, England and Spain^51^.

The chronic disease most commonly addressed was diabetes (*n* = 29, 42.0%), with 16 studies sampling type 2 diabetes (T2DM) population in 19 articles^30–33,42,48,49,52–63^, 10 focusing on type 1 diabetes (T1DM)^51,64–72^ and three enrolling both T1DM and T2DM patients in four articles^45,73–75^. Thirteen studies (18.8%) targeted cardiovascular diseases, with five focusing on heart failure^35,36,76–78^ and the remaining on cardiovascular diseases (CVDs)^34,38,39,79–84^. An equal number addressed chronic respiratory diseases (*n* = 13, 18.8%), with seven studies tackling asthma^41,85–90^ and six focusing on chronic obstructive pulmonary disease (COPD)^91–96^. Lastly, ten studies focused on cancer (*n* = 10, 14.5%)^40,43,44,46,47,50,97–100^. One study separately addressed T2DM and CVDs (either ischaemic heart disease or heart failure)^101^, while two others included both uncontrolled hypertension and diabetes^37,102^ and one focused on T2DM and/or hypertension^103^. Their sample sizes ranged from 18^35^ to 519^101^ and the median size was 94, with 23 studies (33.3%) totalling between 50 and 100 participants and only six studies with more than 200 individuals^33,39,59,88,90,101^.

The vast majority were either individually randomised parallel-group trials (*n* = 52, 75.4% of total studies) or pilot RCTs (*n* = 12, 17.4%), while the remaining five had a cluster-randomised (*n* = 3, 4.3%)^31,37,90^ or an individual cross-over design (*n* = 2, 2.9%)^69,72^.

Out of the 69 studies, 60 were two-arm, while eight (11.6%) had three and one (1.4%) had four groups^31^. Among those with multiple intervention groups, several studies, along with the standard app version, included a different arm with an enhanced intervention, on top of app use, with the possibility of using either the teleconsultation option^64^, Health Counselling intervention^53^, classroom-based programmes^33^, decision support^31^, physician review^100^ or additional app features^84^. Frias et al., instead, had two intervention arms using the same app, but with different follow-ups^37^.

In the control group, most participants received care as usual. However, some studies included lighter technological features for the control group: Johnston et al. provided the control with a simplified drug adherence e-diary installed on phones^83^, while other studies either included basic versions of the app^68^ or administered education/information programmes over mobile phones^35,43^. Ryan et al.^88^ provided the control with enhanced clinical care to exclude attribution of potential benefits to intense interventions in the app group. Franc et al.’s study^60^, besides the Diabeo app arm, included a simpler telemonitoring system via an interactive voice response system, while Kwon et al.^93^ adopted two different exercise regimens.

The follow-ups varied: for 26 studies, they were between 4 and 6 months (37.7%); for 24 studies (34.8%), 1–3 months; in 13 cases (18.8%), >6 months and for the remaining six (8.7%) only 4 weeks.

The majority of the studies (*n* = 44, 63.8%) included internet literacy as an explicit eligibility criterion, as either generic familiarity with mobiles or, more explicitly, ownership of smartphones with specific operating systems. A notable exception is Baron et al.’s study^73^ excluding those with previous mobile telehealth services experience.

Recruitment procedures were mostly traditional, with offline methods for 64 studies (92.7%). Only Morawski et al.^39^ adopted a completely online recruitment strategy, through patient communities, social media, mobile apps and advertisement, while four studies pursued both offline and online strategies, through online advertisement and community forums^40,61,65,82^.

Finally, there were two major approaches regarding the mobile phones in the trial: 36 studies (52.2%) provided participants with study devices after randomisation, while 31 (44.9%) adopted a bring your own device approach, downloading the app on the participant’s smartphone. Only two studies adopted a mixed strategy, opting to install the app on a loaned phone if the participant was incompatible with the software version^35,87^. Key study characteristics are summarised in Table 1, while a per-study overview of selected study characteristics can be found in Supplementary Table 1.

**Table 1:** Key characteristics of included studies (*n* = 69).

Study characteristics	*n* (%)
Disease area	
Diabetes	29 (42.0%)
Cardiovascular diseases	13 (18.8%)
Respiratory diseases	13 (18.8%)
Cancer	10 (14.5%)
Multiple	4 (5.8%)
Publication year^a^	
2008–2013	13 (17.6%)
2014–2016	25 (33.8%)
2017–2019	36 (48.6%)
Country of study	
United States of America	13 (18.8%)
China	7 (10.1%)
Australia	5 (7.2%)
Canada	5 (7.2%)
The Netherlands	5 (7.2%)
United Kingdom	5 (7.2%)
Finland	3 (4.3%)
South Korea	3 (4.3%)
Switzerland	3 (4.3%)
Others	20 (29.0%)
Design	
RCT	52 (75.4%)
Pilot RCT	12 (17.4%)
Cluster RCT	3 (4.3%)
Cross-over RCT	2 (2.9%)
Study arms	
Two	60 (87.0%)
Three	8 (11.6%)
Four	1 (1.4%)
Sample size	
Median (range)	94 (18–519)
<50	14 (20.3%)
51–100	23 (33.3%)
101–150	15 (21.7%)
151–200	11 (15.9%)
>200	6 (8.7%)
Follow-up interval	
Up to 1 month	6 (8.7%)
1–3 months	24 (34.8%)
4–6 months	26 (37.7%)
More than 6 months	13 (18.8%)
Recruitment strategy	
Offline	64 (92.8%)
Online	1 (1.4%)
Mixed	4 (5.8%)
Internet literacy as an eligibility criterion	
Yes	44 (63.8%)
No	25 (36.2%)
Device strategy	
Study device approach	36 (52.2%)
Bring your own device approach	31 (44.9%)
Mixed approach	2 (2.9%)

^a^The total number of included publications is equal to 74.

#### App design and development considerations

Five development factors were analysed comparatively across all studies. Based on the study conducted by Adu and colleagues^24^, we considered the following factors: (i) supporting behavioural theory, (ii) user involvement in the design, (iii) healthcare professional (HCP) involvement in the design, (iv) data security and privacy considerations, (v) pilot testing, which includes all forms of interaction with target users to enhance the usability, acceptability and reliability of the app before its complete testing (full details in Supplementary Table 2).

Regarding health behavioural theories, only a minority of the studies (*n* = 11, 15.9%) reported that the interventions were based on theories and models of behaviour change and were beneficial in developing apps^35,42,43,47,52,57–59,61,73,90^.

Concerning user involvement in app design, 21 studies (30.4%) explicitly mentioned patient engagement strategies, incorporating patient inputs in app design. While most simply cite an interactive approach in close collaboration with intended users, a few specify the process in greater detail. Ryan et al. based their formative work on qualitative interviews of ten asthma patients and two research staff to identify technological adjustments for improved solutions^88^, while other publications included user requirements through survey results^93,99^, iterative piloting^95^ or a consecutive series of user studies during a 26-month development phase^61^. A similar number also engaged HCPs in the app design phase (*n* = 18, 26.1%). While many studies adopted engagement strategies in design involving both users and HCPs, there were a few exceptions. Quinn et al.’s studies included only endocrinologists and Certified Diabetes Educators in design^30,31^, while Yang et al. adopted a Delphi Method with two consultation rounds involving 30 clinical and nursing experts^50^ and Greer et al. reported extensive user testing by a team of clinical researchers^43^. On the contrary, the Few Touch app was developed using focus groups, interviews, feasibility testing and questionnaires over a 3-year period with T2DM patients only^53^.

Data security and privacy information were documented by 33 of the studies (47.8%), mostly pertaining to secure data transfer and storage from app to study servers, the minimisation of medical/personal information stored on the mobile and obtained through the app, and the password protection and encryption of core data. A couple of studies mentioned compliance with the Health Insurance Portability and Accountability Act^30,31,38^, while Wayne et al. mentioned that the Connected Wellness Platform to collect data exceeded Canadian privacy standards for software carrying health information^55^. Only one study performed a comprehensive risk analysis before the trial to ensure data privacy and security^53^.

The incorporation of pilot testing into app development was cited by 28 of the studies (40.6%), with eight additional trials (11.6%) characterised as pilot RCTs themselves. Of those 28 studies, the great majority reported observational single-arm studies instrumental in assessing study feasibility and usability. Some studies instead documented multiphase pilot testing: Vorrink et al. reported three rounds of pilot testing initially starting with healthy volunteers and later including COPD patients^95^, while Kearney et al. developed and validated the advanced symptom management system through a two-arm pilot study in Scotland with ten patients receiving chemotherapy and a feasibility study to evaluate the system’s acceptability on a convenience sample of 18 patients and nine HCPs^98^. Boer et al.’s Adaptive Computerised COPD Exacerbation Self-management Support tool was validated through a 3-month prospective observational study, but was further optimised during the trial with a 2-week run-in period to familiarise participants with the technology^96^. Finally, a few studies that did not test apps specifically designed for research purposes, but rather selected commercially available ones adopted different forms of piloting, by identifying software with the highest usability ratings by users or researchers adopting the Mobile App Rating Scale^39,40,84^. The developmental factors adopted are summarised in Table 2.

**Table 2:** Developmental factors considered by the included studies (*n* = 69).

Name	Articles
Data security and privacy considerations	33 (47.8%)
Healthcare professional involvement in the design	18 (26.1%)
Pilot testing	28 (40.6%)
Supporting behavioural theory	11 (15.9%)
User involvement in the design	21 (30.4%)

#### Features of the mHealth interventions

The interventions differed significantly in content, level of HCP involvement and degree of automation in decision support (Supplementary Table 3).

Regarding professional participation, 26 studies (37.7%) excluded any in excess of that guaranteed to control patients during routine follow-up visits. Among these, seven also embedded a low technology-automation level in decision support: three studies aimed at sharing information and increasing disease awareness through the app^58,82,97^, while others either worked as digital diaries allowing parameter recording under clinical supervision without direct intervention^67,69^, simulated e-therapy through multimedia contents^43^ or delivered mindfulness training^40^. The remaining 19 studies adopted more intensive automated technology: four recorded participants’ medication adherence through reminders^39,63,84^ or an artificial intelligence platform with dosing instructions^38^; four others fostered improved physical activity automatically selecting the appropriate intensity progression for exercise regimens^61,93,99^ or providing feedbacks on trunk control^79^, while the remaining 11 delivered automated advice or feedback about the data entered in the app based on decision-support systems or automated algorithms, with minimised involvement of HCPs^35,41,59,68,71,78,83,87,94,96,103^.

On the other hand, 43 studies (62.3%) tested app interventions including different forms of HCP involvement, which typically engaged either clinicians or nurses. Among these, 23 did not pair the HCP intervention with additional technological decision support: one study facilitated therapy optimisation by providers based on adherence overview^37^; two studies included HCP involvement in classroom-based programmes^33^ or group sessions^42^ as co-interventions to a lifestyle changing app with no automated feedbacks; two others supported professional monitoring of participants’ physical activity with the possibility of adjusting goals^95^ or reinforcing adherence^92^; four enabled direct patient–physician communication through forums or real-time consultations^44,46,47,70^, while the remaining 14 transferred data to dedicated professionals. A further distinction between this latter group of 14 studies can be made between studies where the data input was used by HCPs to provide personalised feedbacks during visits or share recommendations through text messages with a predefined frequency^48,53,65,72,75,80,100,101^, and those where data transferred through the app continuously triggered study coordinator intervention whenever the responses suggested a deteriorating health^36,45,55,73,85,90^.

The final 20 studies included both HCP involvement and higher-intensity technology automation. Some of the app interventions calculated and suggested optimal insulin doses under general professional oversight and with facilitated patient–physician communication^51,56,64,66^, while the remaining provided automatic machine-based feedbacks as a first-level support with the possibility for professionals to intervene if warranted^30,31,34,49,50,52,57,60,76,77,86,88,89,91,98,102^.

Only five studies provided HCP with a mobile app coupled with the patients’ ones. In Cingi et al.’s study, physicians could view all patients’ inputs and respond to messages and broadcast multimedia to participants through their app^85^. Similarly, in other studies, a clinician app was associated with patient data^34,45^ or enabled patient–physician communication^44,55^.

**Fig. 2:**
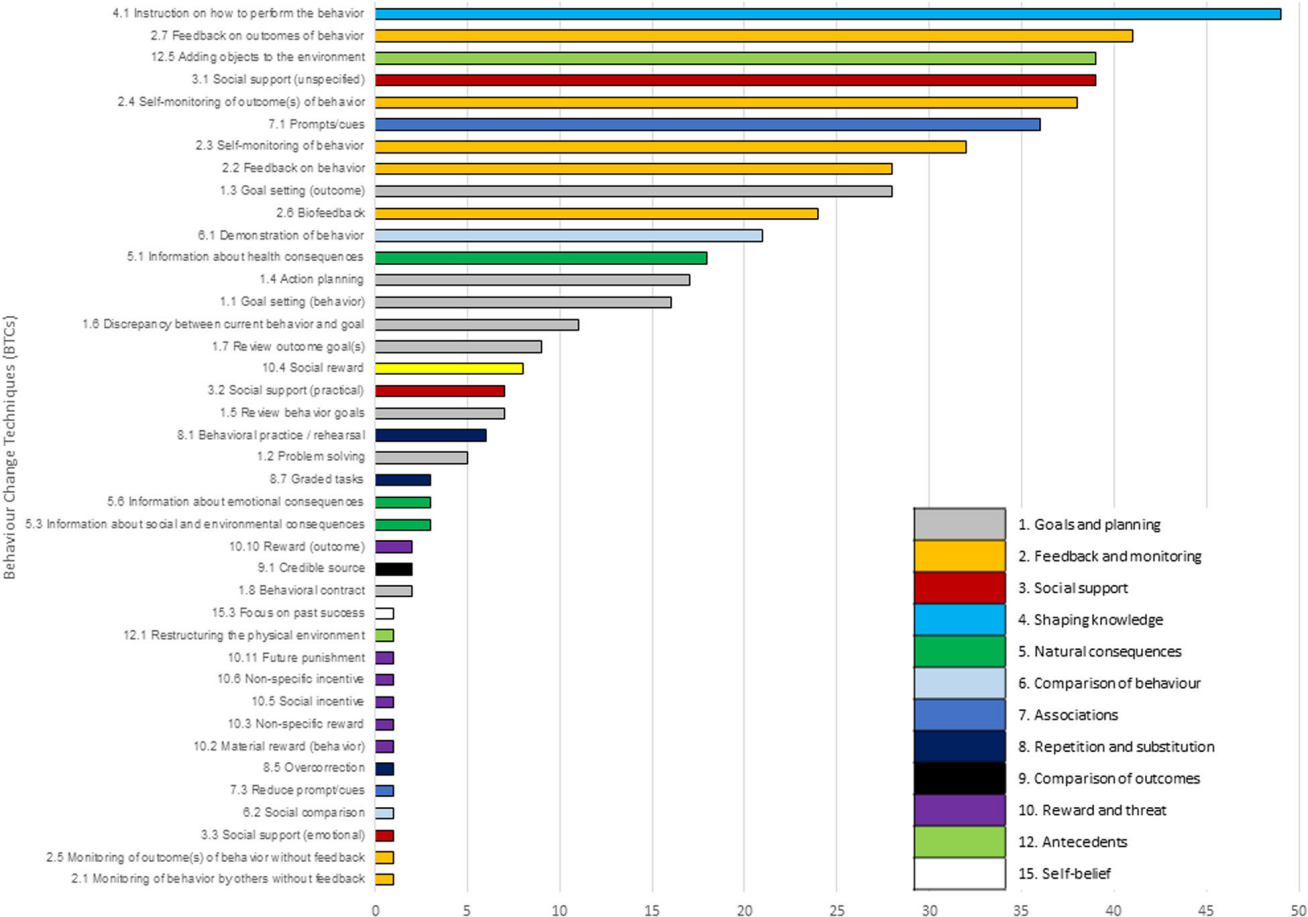
BCT components adopted. Behaviour Change Techniques components and related clusters included in the selected studies (*n* = 69).

Regarding the behaviour change techniques (BCTs) adopted in the studies^104^, the median number of BCT categories identified was eight (mean 7.46, SD 3.02). Twenty interventions (29.0%) included up to five BCTs, 37 had between five and ten BCTs, while the remaining 12 (17.4%) had more than ten BCTs. The maximum identified BCTs was 14^67,68^, while the most common individual BCTs were: ‘Instruction on how to perform a behaviour’ (*n* = 49, 71,0%), ‘Feedback on outcomes of behaviour’ (*n* = 41, 59.4%), ‘Social support’ (*n* = 39, 56.5%), ‘Adding objects to the environment’ (*n* = 39, 56.5%), and ‘Self-monitoring of outcome(s) of behaviour’ (*n* = 38, 55.1%) (Fig. 2). Of the 93 possible BCTs identified in the taxonomy, 40 were used in at least one study and 14 in at least 14 (20%) studies.

#### Primary outcomes

Primary outcomes were explicitly identified in 56 studies (where a formal power calculation was performed) and were classified based on the taxonomy for outcomes in clinical research^105^. The types of primary outcome measures varied in terms of core areas, although they most frequently pertained to physiological and clinical outcomes (*n* = 29, 51.8% of the total primary outcomes identified in the studies), followed by life impact (*n* = 23, 41.1%), resource use (*n* = 3, 5.4%) and adverse events (*n* = 1, 1.8%).

Regarding specific outcome domains, endocrine outcomes were the most commonly reported, haemoglobin A1c levels (*n* = 20), with only seven studies reporting significant differences between groups at follow-up^31,45,57,60,64,65,70^. Among the remaining studies, seven analysed the impact on physical functioning and seven on delivery of care, both belonging to the life impact domain. Regarding physical functioning, three studies evaluated physical activity in terms of steps/day^61,94,95^, while two assessed the impact on disease-specific self-care^76,78^ and two analysed the change in respiratory function parameters through the 6-min walking test^93,99^. Statistically significant improvements were observed only in two studies^61,78^. With regard to care delivery, six studies assessed the impact on medication adherence, through the administration of either a self-reported rating scale^39,63,84,90^, a composite score^83^ or pill count^36^, while the remaining study evaluated a cardiac rehabilitation programme^80^. Of these, five studies demonstrated significant outcome improvements^39,63,80,83,84^. All the reported primary outcome domains classified by a core area and the statistical significance of the trial results (with *p* value ≤0.05) are shown in Table 3, with additional information in Supplementary Table 4.

**Table 3:** Primary outcome domains and statistical significance (*α* ≤ 0.05) of the included studies (*n* = 56).

Core area	Outcome domain	Positive results	Neutral results	Negative results
II. Clinical outcomes	3. Cardiac outcomes	3	0	0
	5. Endocrine outcomes	7	13	0
	9. General outcomes	1	0	0
	14. Metabolism and nutrition outcomes	1	0	0
	22. Respiratory, thoracic and mediastinal outcomes	0	4	0
III. Life impact	25. Physical functioning	2	5	0
	28. Emotional functioning/wellbeing	2	1	0
	29. Cognitive functioning	1	0	0
	30. Global quality of life	2	3	0
	32. Delivery of care	5	2	0
IV. Resource use	34. Economic	0	1	0
	35. Hospital	0	1	0
	27. Societal/care burden	1	0	0
V. Adverse events	38. Adverse events/effects	0	1	0

### Risk-of-bias assessment

Sixty-four (93%) studies presented an overall high risk of bias per the Cochrane RoB 2 tool, whilst only five had a low risk for at least four domains^53,61,64,70,82^. The main issue was the plausible impossibility to blind study participants to the intervention, together with potential deviations from the intended interventions. The randomisation process and the selection of the reported results were the domains showing the lowest bias risk. The detailed, individual risk-of-bias analysis is provided in the Supplementary information materials (Supplementary Fig. 1).

## Discussion

We systematically reviewed the randomised studies on the impact of mhealth apps on four NCDs. We identified 69 studies (74 papers), published since 2008 from 24 countries, focusing on diabetes (*n* = 29), CVDs (*n* = 13), chronic respiratory diseases (*n* = 13), cancer (*n* = 10) or combinations of these.

The mHealth app impact was assessed on a wide range of primary outcomes. Per Dodd et al.’s taxonomy^105^, they most frequently pertained to endocrine, cardiac or respiratory clinical outcomes (*n* = 27, e.g. glycated haemoglobin and systolic blood pressure), but also physical, emotional or cognitive functioning (*n* = 11), care delivery (*n* = 7, e.g. medication adherence) and global quality of life (*n* = 5). Although a quantitative synthesis was not possible in this review due to the broad study aims and the subsequent heterogeneity of the studies, we noted inconclusive significance for many of them across chronic diseases. Statistically significant, improved primary outcomes were reported in 26 studies (eight on endocrine outcomes, five on delivery of care outcomes and three on cardiac outcomes, among others). These results confirm the widespread concerns in the scientific literature about the inability to couple the abundant production of mHealth apps with adequate vetting processes to generate evidence for their adoption^106–108^.

This review focused on two main cornerstones that help explain the inadequacy of current peer-reviewed randomised studies of mHealth apps and could be leveraged to improve future ones.

On one side, there are important considerations related to app development, before the actual use in clinical studies. Our review evaluated several developmental factors for possible adoption in the design stage, all of which were inadequately recorded in the majority of the studies.

Despite the criticality of data security and privacy issues, often unaddressed with health apps^109,110^, only 33 studies documented these aspects, even if preliminarily, in the trial report. Similarly, only about a third of them reported user and HCP engagement during the app design, through basic or more articulated (e.g. Delphi process) strategies. Although our findings show some improvements, vis à vis a previous review on diabetes self-management apps^24^, more effort is needed to assign end-users a pivotal role in app development through the incorporation of their needs, expectations and experiences^111^. Furthermore, only 28 (45.9%) of the non-pilot RCT studies mentioned previous pilot testing of the intervention, which seems a missed opportunity to prove a new intervention’s viability, manage its risk and identify any deficiencies before substantial resource commitment. Finally, few interventions (11, 15.9%) were grounded in behavioural change theory. This shortcoming is currently a key missing element for digital health tools to achieve sustained behaviour change^112^ and could be related to software developers overlooking important components for robust development and evaluation^113^.

These critical steps in the app development process gain further relevance considering that few mHealth solutions, among those investigated in RCTs, progress to wide availability in routine practice and major stores^114^, thus emphasising the paradox of wide adoption of apps untested in clinical research, while clinically investigated apps rarely scale up to real-world adoption.

As for HCP involvement, this review found 43 studies (62.3%) with additional human-led components. The support or encouragement provided to the patient by someone, whether it is directed at praising or rewarding behaviour or supporting self-management could decisively impact the intervention. A previous meta-analysis identified that, compared with diabetes apps with low-frequency HCP feedback, those with high-frequency feedback had a significant effect^14^. In the present review, studies involving additional human-led components showed no higher likelihood of a positive effect on outcomes for individuals in the intervention arm.

In addition, app-based interventions can differ greatly in their content and in the way they induce behaviour change. A median of eight BCTs was tapped by each mHealth intervention, with significant variability among them (SD 3.03). In apps containing gamification strategies, which share significant overlap with health BCTs and draw upon leader boards, prizes and rewards to motivate individuals^115^, the median rises to 14 BCTs with consistent inclusion of the ‘reward and threat’ category (81% apps)^116^. Gamification is indeed a significant factor to acknowledge in the development of mHealth apps for its promise to drive user behaviour and increase engagement through game elements, and ad hoc models have been proposed to support designers of gamified, condition-oriented solutions^117^. However, the adoption of gaming components was infrequent in the apps for chronic NCDs in this review, with only 12 (17.4%) studies implementing ‘reward and threat’ techniques. The most common behaviour change categories were instead ‘Feedback and monitoring’ (91%) and ‘Shaping knowledge’ (72%). High prevalence of self-regulatory techniques and, to a lower extent, prompts such as alerts and reminders is coherent with previous studies^118,119^, although these adopted an earlier 26-category taxonomy version^120^ and specifically aimed at promoting physical activity. A striking difference from earlier reviews is the frequent incorporation of the BCT ‘Adding objects to the environment’, highlighting the ambivalence between the adoption of personal and study devices in intervention delivery. When an ad hoc device was provided after randomisation, an extra object was added in the environment to support the desired behaviour, and the related BCT was hence recorded. Lack of awareness and research on specific combinations of BCTs additionally emphasises the interventions’ poor grounding in behaviour change theories.

Our findings highlight further methodological issues concerning app testing.

A recent review of study protocols available via clinicaltrials.gov examined the clinical evidence underlying digital health interventions, highlighting the studies’ relatively low quality, small size and limited likelihood of being significantly powered to demonstrate treatment effects compared to drugs and traditional medical devices, which mandate stricter regulatory guidelines on safety and efficacy^121^. Although we considered published peer-reviewed reports of interventional studies and not study protocols, our results agree with the previous contribution: relatively short follow-ups—only 13 studies followed up >6 months—and median sample size of 94 patients, with several studies adopting convenience sampling and others not reaching the expected enrolment level or inadequately accounting for drop-out rates in their sample size calculations. Furthermore, 28 were pilot RCTs in need of subsequent investigation^122^. Also, formal risk-of-bias assessment performed according to standard tools revealed a high risk of bias for the majority of RCTs, highlighting the inability of both currently published studies and available assessment metrics to keep up with the specific methodological challenges of mobile apps.

These results should be assessed considering the ongoing literature debate on the evaluation of digital health interventions and mHealth apps. While some reject digital exceptionalism and accept RCTs as the golden standard^123^, others think that digital technologies cannot be held to the same standards as new drugs/devices^124^ and support the adoption of more agile, yet equally robust, methodologies^125^. Given the distinguishing characteristics of mHealth apps^126^, designs other than parallel-group RCTs have been proposed. These include the multiphase optimisation strategy (MOST), the sequential multiple assignment randomised trial (SMART) and the micro-randomised trial^127–130^. This also seems to be the direction embraced by some regulatory bodies such as—for instance—the Federal Institute for Drugs and Medical Devices in Germany (Bundesinstitut für Arzneimittel und Medizinprodukte, BfArM) that recognises the relevance of alternative study designs and methods such as Pragmatic Clinical Trials, SMART or MOST^131^.

Nonetheless, none of these was implemented in the peer-reviewed studies identified, emphasising the preference for parallel-group RCTs or checklists of quality criteria that show significant variability and no clear method for their development^132^.

The review results additionally light up the current debate: with 55% of the randomised studies showing neutral findings—i.e. non-significant difference—it is legitimate to discuss whether results could have differed with more robust development and design of the underlying apps, implementation of agile study designs or appropriate study characteristics of the RCT studies.

### Limitations

There are several limitations of the current review. A meta-analysis was impossible due to the heterogeneity of studies explored. Non-English papers were excluded. We reviewed randomised studies only, which allowed focusing on the theoretically more robust evidence to assess the impact of mHealth apps in chronic disease while excluding other potentially relevant evidence sources. Furthermore, the taxonomy adopted for the BCT analysis was not specifically developed for mHealth apps, inevitably entailing authors’ judgement. Finally, the coding was based on the information in the study report and related sources. Some of the features may have been untracked, possibly underestimating the number of BCTs tapped.

## CONCLUSIONS

This systematic review sheds light on how design and development processes, choice of outcomes and behavioural change theory underpinning mHealth apps are fundamental in determining the value of digital interventions.

Furthermore, based on exploratory quantitative analyses of the association between study characteristics and a positive statistically significant result, our study seems to suggest a relatively lower success among apps for respiratory conditions (asthma, COPD) compared to CVDs, oncology or diabetes. This finding must be confirmed in future studies, but certainly highlights how the evidence on effective or cost-effective apps is not generalisable *tout court* across different health conditions. The widely adopted concept of ‘equivalence’ used in medical devices regulation systems must be carefully considered in the case of mHealth apps^133,134^. Another interesting finding is the lower proportion of positive studies among those with longer (≥6 months) follow-up. This may signal issues with sustained adherence and engagement with the intervention and calls for research intended to identify strategies that maintain higher levels of engagement of end-users and successful retention rate in the long run.

Finally, among four different outcome areas, life impact outcomes seem to report more positive results compared to clinical outcomes, resource use or adverse events. This finding draws attention to the importance of the most appropriate outcome selection in the evaluation of mobile app interventions.

However, especially in view of the recently adopted EU Regulation of Medical Devices and the upcoming EU Regulation on Health Technology Assessment^135,136^, follow-up research is urgently needed to further explore these elements that deserve deeper attention, confirm these initial hypotheses and to better investigate the impact of BCTs on the effectiveness of mHealth apps.

Developers, methodologists and trialists are encouraged to carefully consider these additional elements when designing or running clinical evaluations of apps, to elucidate whether trial results reflect their true benefits or a biased estimate due to unsuitable designs or suboptimal implementation of the intervention.

Acknowledging the specific features of health apps (e.g. security issues, continuous content and software updates, and the impossibility of blinding participants and investigators)^126^ is necessary to support policy-makers, providers and developers in their large-scale evaluation and consistent and effective deployment for chronic disease management.

The COVID-19 pandemic may have favoured large-scale adoption of digital tools^137^, but only through sustained and appropriate evidence generation they will shape successful care models and significantly contribute to CD management.

## Methods

### Information sources

This systematic review was guided by the PRISMA statement and checklist^138^.

A literature search strategy was developed using keywords and MeSH (Medical Subject Headings) related to mHealth apps and pertinent chronic diseases. The full search strategy was first defined on Medline (Supplementary Note 1) and then adapted to the other engines. The search was performed on Medline (OVID interface), ScienceDirect, the Cochrane Library and Scopus, and last updated on 23 November 2019. To ensure literature saturation, we scanned the reference lists of the studies and systematic reviews and meta-analyses initially retrieved by the search or recently completed on PROSPERO and added additional publications of interest to the review. The review was not registered. A detailed protocol was prepared and circulated among the project team.

### Eligibility criteria

The search strategy targeted trials with a randomised design involving individuals with one of the four main NCDs as identified by the World Health Organisation—cardiovascular diseases, cancers, chronic respiratory diseases and diabetes^139^—and the use of a mobile health app, a small, self-contained software coded for a specific purpose and usually optimised to be downloaded and run on mobile phones and tablets^140^. In terms of study design, we selected any type of randomised trials (parallel-group trials, cluster-randomised trials and cross-over trials, and other recent study designs specifically developed for digital technology). Concerning the target population, no further restriction criteria based on patient characteristics, such as age, gender, ethnicity or employment status were applied. All possible comparators were deemed relevant, including current standards of medical care as well as other eHealth interventions, such as telephone follow-up, text messaging or a simplified version of the app intervention. All quantitively measured outcomes were included and classified based on Dodd et al.’s taxonomy^105^. In terms of publication status, only peer-reviewed journal articles published in English after 2008 (when the App Store and PlayStore were first opened) were eligible. We excluded studies where the app was only used by HCPs or that focused on feasibility only. Further details about all inclusion and exclusion criteria are provided in Supplementary Table 5.

### Study selection

Studies identified via database searches were first screened by title and abstract by two independent reviewers (F.P. and M.C.). Duplicates were excluded and, in case of disagreement, a third independent reviewer was consulted (R.T.). For studies meeting the inclusion criteria, or when unclear, the full article was retrieved. The same reviewers assessed the full-text papers to determine inclusion based on the stated eligibility criteria. For studies deemed ineligible for inclusion at the full-text analysis stage, the reasons for exclusion were recorded. Cohen’s kappa was calculated to determine the inter-rater agreement between the reviewers.

### Data collection and extraction

We used a pre-piloted data extraction form elaborating the CONSORT-EHEALTH checklist extension^141^ and the Cochrane Consumers and Communication Review Group’s data extraction template^142^. Information was extracted from each study on: (i) study design and participant characteristics; (ii) app design and development; (iii) features of the mHealth intervention, including embedded BCTs^104^; (iv) types of outcome measures and reported results.

### Risk-of-bias assessment

Since no official methodological standards are available for empirical studies on mobile apps, we used the Revised Cochrane risk-of-bias tool for randomised trials (RoB 2)^143^.

Assessment of risk of bias is regarded as an essential component of systematic reviews on the effects of an intervention. An evaluation of the risk of bias in each study included in the systematic review documents potential flaws in the evidence summarised and contributes to the certainty in the overall evidence. The RoB 2 tool provides a framework for assessing the risk of bias in a single estimate of an intervention effect reported from a randomised trial for a specific outcome or endpoint. This revised Cochrane tool was published in July 2019 and is structured into five different bias domains (i.e. risk of bias arising from the randomisation process, risk of bias due to deviations from the intended interventions (effect of assignment to intervention/effect of adhering to intervention), missing outcome data, risk of bias in the measurement of the outcome, risk of bias in the selection of the reported result). The assessment of each domain eventually leads to one of the following three judgements: low risk, some concerns or high risk. An overall risk of bias judgement, based on the five dimensions introduced before, is given to judge the overall confidence in the result. We assessed the treatment effect on the primary outcome or, when the primary outcome was not mentioned explicitly or could not be identified indirectly through sample size calculations, on the first outcome reported in the results^144^. One co-author performed the quality assessment of studies (F.P.), and a second co-author independently double-checked the assessment (O.C.).

### Data synthesis

Given the broad scope of the review, considerably high heterogeneity in terms of the type of NCDs, study characteristics, developmental factors, intervention structure and outcomes observed were anticipated. Therefore, the preferred approach to summarising the data was through a narrative synthesis, tabulation and descriptive analysis of all items extracted from the studies.

## Supplementary information

Supplementary Information

## Data Availability

All data included in this study are available within the paper and its Supplementary information. Source data are available from the corresponding author upon request.
