# Supplementary material for: Development features and study characteristics of mobile health apps in the management of chronic conditions: a systematic review of randomised trials
Source: NPJ Digit Med. 2021 Oct 5;4:144. doi: 10.1038/s41746-021-00517-1 (PMC8492762; doi:10.1038/s41746-021-00517-1)
Supplement: Supplementary file 1 — Supplementary Information [file 41746_2021_517_MOESM1_ESM.pdf]

Supplementary Information

Supplementary Figure 1 *- Graphical display of the results of the risk of bias analysis (n = 69)*

	Domain 1	Domain 2	Domain 3	Domain 4	Domain 5	Overall
**Agarwal et al. 2019**	+	?	?	+	?	-
**Alanzi et al. 2018**	?	?	?	?	?	-
**Athilingam et al. 2017**	?	-	-	?	?	-
**Baron et al. 2017**	+	-	+	+	+	-
**Bee et al. 2016**	+	-	-	+	+	-
**Boer et al. 2019**	+	?	?	?	+	-
**Burckhardt et al. 2018**	+	?	-	?	+	-
**Castensoe et al. 2018**	+	?	+	+	+	?
**Charpentier et al. 2011**	+	+	+	+	?	?
**Cingi et al. 2015**	+	-	?	?	?	-
**Dang et al. 2017**	+	?	?	?	?	-
**Drion et al. 2015**	+	?	+	?	?	-
**Egbring et al. 2016**	+	?	+	?	+	-
**Eyles et al. 2017**	+	?	+	+	+	?
**Farmer et al. 2017**	+	+	?	?	+	-
**Foley et al. 2016**	+	-	+	?	?	-
**Forjuoh et al. 2014**	?	?	-	-	+	-
**Franc et al. 2019**	+	?	?	+	+	-

	Domain 1	Domain 2	Domain 3	Domain 4	Domain 5	Overall
Frias et al. 2017	?	?	?	+	+	-
Goyal et al. 2018	?	?	+	+	+	-
Grady et al. 2017	?	-	?	+	+	-
Greer et al. 2019	+	?	+	?	+	-
Hagglund et al. 2015	?	-	+	?	+	-
Hochsmann et al. 2019	+	?	+	+	+	?
Huang et al. 2019	-	?	?	?	?	-
Ji et al. 2019	?	-	-	?	?	-
Johnston et al. 2016	?	-	+	?	?	-
Karhula et al. 2015	+	?	?	?	+	-
Kearney et al. 2009	+	?	?	?	?	-
Kim et al. 2016	?	?	-	?	?	-
Kirwan et al. 2013	?	?	?	+	+	-
Klee et al. 2018	+	-	-	+	+	-
Kleinman et al. 2017	+	?	?	+	+	-
Kosse et al. 2019	?	-	+	?	+	-
Kwon et al. 2018	+	-	-	?	+	-
Labovitz et al. 2017	?	-	+	?	+	-
Liu et al. 2016	?	-	?	+	?	-
Logan et al. 2012	+	?	+	+	?	-

	Domain 1	Domain 2	Domain 3	Domain 4	Domain 5	Overall
Lyu et al. 2016	+	-	?	+	+	-
Moore et al. 2014	?	?	+	+	?	-
Morawski et al. 2018	+	?	?	?	+	-
Or et al. 2016	+	?	+	+	?	-
Orsama et al. 2013	+	-	+	+	?	-
Quinn et al. 2008	?	-	?	+	?	-
Quinn et al. 2011	+	?	+	?	+	-
Rosen et al. 2018	+	?	?	?	+	-
Rossi et al. 2010	+	?	+	+	?	-
Rossi et al. 2013	+	?	?	+	+	-
Ryan et al. 2012	+	?	?	?	?	-
Santo et al. 2018	+	?	?	?	+	-
Seto et al. 2012	+	?	?	?	+	-
Shin et al. 2016	+	?	+	+	?	-
Skrovseth et al. 2015	+	?	?	+	+	-
Stukus et al. 2018	?	?	+	+	+	-
Sun et al. 2017	?	-	-	?	+	-
Sun et al. 2019	+	-	-	+	+	-
Tabak et al. 2013	+	-	+	+	+	-
Torbjorsen et al. 2014	+	?	+	+	+	?

	Domain 1	Domain 2	Domain 3	Domain 4	Domain 5	Overall
**Varnfield et al. 2014**	+	?	-	-	-	-
**Vorrink et al. 2016**	+	?	-	?	?	-
**Vuorinen et al. 2014**	?	?	+	?	+	-
**Wang et al. 2014**	?	?	+	+	?	-
**Wang et al. 2018**	+	?	+	+	?	-
**Wang et al. 2019**	+	-	-	?	?	-
**Wayne et al. 2015**	+	?	?	+	+	-
**Yang et al. 2019**	+	-	+	?	?	-
**Zairina et al. 2016**	+	?	+	?	+	-
**Zhou et al. 2016**	?	?	-	+	+	-
**Zhu et al. 2018**	+	?	+	?	?	-

Domains:
Domain 1: Bias arising from the randomization process
Domain 2: Bias due to deviations from intended intervention
Domain 3: Bias due to missing outcome data
Domain 4: Bias in measurement of the outcome
Domain 5: Bias in selection of the reported result

Judgement:
- High
? Some concerns
+ Low

Supplementary Table 1 – Characteristics of included studies (n = 69)

Author year	Target condition	Study aims	Country of study	Design	Study arms	Sample size	Follow-up interval	Device strategy
**Agarwal 2019**	T2 DM	To conduct a pragmatic RCT of the BlueStar mobile app on T2DM patients with poorly controlled blood sugar to determine if use of the app leads to improved HbA1c levels through better self-management	Canada	RCT	2	223	6 months	Study device
**Alanzi 2018**	T2 DM	To evaluate the use of WhatsApp for increasing knowledge, self-efficacy, and awareness about diabetes management	Saudi Arabia	RCT	2	92	8 weeks	Personal device
**Athilingam 2017**	Heart failure	To test the feasibility of a newly developed mobile app in improving self-care behaviors and quality of life of patients with heart failure	USA	Pilot RCT	2	18	4 weeks	Mix
**Baron 2017**	T1 DM<br>T2 DM	To examine the effects of a mobile telehealth intervention on a range of clinical and patient-reported outcomes	UK	RCT	2	81	9 months	Study device
**Bee 2016**	T2 DM	To investigate app feasibility to deliver an insulin titration algorithm in insulin-naive T2 DM patients	Singapore	Pilot RCT	2	66	24 weeks	Personal device
**Boer 2019**	COPD	To test an innovative mobile tool which aims to tailor self-management support more efficiently than a written action plan, without heavily increasing the involvement of health care professionals	The Netherlands	RCT	2	87	12 months	Study device
**Burckhardt 2018**	T1 DM	To investigate the impact of continuous glucose monitoring with remote monitoring on psychosocial outcomes in parents of children with T1DM	Australia	Crossover RCT	2	49	3 + 3 months (2-month washout period in-between)	Personal device
**Castensoe 2018**	T1 DM	To test whether an app can improve self-management among young people with T1 DM	Denmark	RCT	2	151	12 months	Personal device
**Charpentier 2011**	T1 DM	To evaluate the efficiency of the Diabeo system in improving metabolic control of poorly controlled T1 DM	France	RCT	3	180	6 months	Study device
**Cingi 2015**	Asthma	To measure the impact of a mobile patient engagement app on health outcomes in asthma patients	Turkey	RCT	2	136	3 months	Personal device
**Dang 2017**	Heart Failure	To test if daily monitoring using a mobile phone-based system would improve patient self-care efficacy, knowledge, and QoL	USA	RCT	2	61	3 months	Study device
**Drion 2015**	T1 DM	To investigate the effects of a mobile app on QoL for patients with T1 DM	The Netherlands	RCT	2	63	3 months	Personal device
**Egbring 2016**	Breast cancer	To explore the impact of a mobile and web-based app in both an unsupervised setting and a setting supervised by the treating physician	Switzerland	RCT	3	139	6 weeks	Personal device
**Eyles 2017**	CVD	To determine the effectiveness of an app to support people with CVD to make lower-salt food choices	New Zealand	RCT	2	66	6 weeks	Personal device
**Farmer 2017**	COPD	To determine the efficacy of a tablet computer-based system of monitoring, in improving quality of life and clinical outcomes for patients with moderate to very severe COPD	UK	RCT	2	166	12 months	Study device
**Foley 2016**	Breast cancer	To develop an app and test whether having access to additional information would lower anxiety and depression scores in breast cancer patients	Ireland	Pilot RCT	2	39	2 weeks	Study device
**Forjouh 2014**	T2 DM	To test the effectiveness of two different diabetes self-care interventions on glycemic control	USA	RCT	3	376	12 months	Study device

Author year	Target condition	Study aims	Country of study	Design	Study arms	Sample size	Follow-up interval	Device strategy
**Franc 2019**	T2 DM	To investigate if app could help subjects with T2D reach glycemic targets during basal insulin initiation and titration	France	RCT	3	189	13 months	Study device
**Frias 2017**	Uncontrolled hypertension + T2 DM	To assess the impact on blood pressure and HbA1c using a DMO including digital medicines with ingestible sensor, a wearable patch, and a mobile device app	USA	Pilot Cluster RCT	3	118	12 weeks	Study device
**Goyal 2018**	T1 DM	To design, develop, and evaluate an app aimed to assist adolescents with the self-management of T1 DM	Canada	RCT	2	92	12 months	Study device
**Grady 2017**	T1 DM<br>T2 DM	To assess changes in glycemic control using an app in conjunction with a wireless blood glucose meter	UK	RCT	2	137	24 weeks	Study device
**Greer 2019**	Various cancer types	To test the efficacy of a mobile app intervention in reducing anxiety in patients with incurable cancer and elevated anxiety	USA	RCT	2	154	12 weeks	Study device
**Hagglund 2015**	Heart Failure	To evaluate whether a new home intervention system based on a tablet computer had an effect on self-care behaviour	Sweden	RCT	2	82	3 months	Study device
**Hochsmann 2019**	T2 DM	To investigate if the BCT–based smartphone game can motivate inactive individuals with T2 DM for regular use and thereby increase their intrinsic PA motivation	Switzerland	RCT	2	36	24 weeks	Personal device
**Huang 2019**	T2 DM	To determine the feasibility, effectiveness, acceptability, and clinical outcomes of using a smartphone app to improve medication adherence in a multiethnic Asian population with T2 DM through a pilot study	Singapore	Pilot RCT	2	51	12 weeks	Personal device
**Ji 2019**	Lung Cancer	To examine the outcome of home-based pulmonary rehabilitation regarding exercise capacity, dyspnea symptoms, and QoL in adult patients treated for NSCLC	South Korea	RCT	2	94	12 weeks	Personal device
**Johnston 2016**	Myocardial infarction	To assess if the use of an interactive patient support tool would improve adherence to antiplatelet treatment	Sweden	RCT	2	174	6 months	Personal device
**Karhula 2015**	T2 DM<br>Heart disease	To assess the benefits of a structured mobile coaching program with a remote monitoring system on QoL among patients suffering from heart disease or diabetes	Finland	RCT	2	519	12 months	Study device
**Kearney 2009**	Various cancer types	To investigate the viability of the trial design and explore any effect of the advanced symptom management system on incidence, severity, and distress of six chemotherapy-related symptoms	UK	Pilot RCT	2	112	5 cycles	Study device
**Kim 2016**	Asthma	To explore the feasibility and efficacy of adopting an app in adult asthma patients visiting an allergic clinic	South Korea	RCT	2	44	8 weeks	Personal device
**Kirwan 2013**	T1 DM	To examine the effectiveness of a free app with text-message feedback from a certified diabetes educator to improve glycemic in adult patients with T1 DM	Australia	RCT	2	72	6 months	Personal device
**Klee 2018**	T1 DM	To evaluate the impact of a multidisciplinary intervention consisting of a mHealth app, educational intervention by specialized nurses and regular insulin dose adaptation by diabetologists on metabolic control of T1 DM	Switzerland	Crossover RCT	2	55	3 months + 3 months	Personal device
**Kleinman 2017**	T2 DM	To test a mHealth platform that supports self-management and facilitates patient–provider data exchange and communication	India	RCT	2	91	6 months	Personal device
**Kosse 2019**	Asthma	To evaluate the effectiveness of the ADAPT intervention in improving ICS adherence in adolescents with asthma	The Netherlands	Cluster RCT	2	253	6 months	Personal device

Author year	Target condition	Study aims	Country of study	Design	Study arms	Sample size	Follow-up interval	Device strategy
**Kwon 2018**	COPD	To develop a home-based mHealth pulmonary rehabilitation program for patients with COPD to improve their daily physical capacity and QoL	Korea	RCT	3	85	12 weeks	Personal device
**Labovitz 2017**	Ischemic stroke	To test the feasibility and impact of using a mobile platform in a stroke population	USA	RCT	2	28	12 weeks	Study device
**Liu 2016**	Asthma	To test if the awareness of daily asthma control status and instant advice through a mobile telephone-based interactive programme would lead to better asthma control	Taiwan	RCT	2	120	6 months	Mix
**Logan 2012**	Uncontrolled hypertension + DM	To test the effectiveness of a telemonitoring system providing self-care messages on diabetic patients' smartphone	Canada	RCT	2	110	12 months	Study device
**Lyu 2016**	Head and neck cancer	To investigate the feasibility and superiority of using WeChat as an assistant to clinical tracking and follow-up of patients with head and neck tumors	China	RCT	2	108	6 months	Personal device
**Moore 2014**	Hypertension	To assess whether a technology-supported apprenticeship in hypertension management would improve blood pressure control compared with a successful coaching model	USA	RCT	2	42	12 weeks	Study device
**Morawski 2018**	Hypertension	To evaluate the association of medication adherence and blood pressure control with a "standalone" smartphone app among patients with poorly controlled hypertension	USA	RCT	2	411	12 weeks	Personal device
**Or 2016**	T2DM<br>Hypertension	To evaluate whether the outcomes of patients with T2DM and/or hypertension who performed tablet-based self-monitoring would improve compared to conventional disease self-monitoring	Hong Kong	Pilot RCT	2	63	3 months	Study device
**Orsama 2013**	T2 DM	To evaluate a mobile telephone-based remote patient reporting system aimed at improving self-management and health status in individuals with T2 DM	Finland	RCT	2	56	10 months	Study device
**Quinn 2008**	T2 DM	To test the feasibility of a cellphone-based diabetes management software system	USA	Pilot RCT	2	30	3 months	Study device
**Quinn 2011**	T2 DM	To test whether adding mobile app coaching and web portals to community primary care would reduce glycated hemoglobin levels in patients with T2 DM	USA	Cluster RCT	4	163	1 year	Study device
**Rosen 2018**	Breast cancer	To explore the efficacy of commercially available mobile app-based mindfulness training delivered to women diagnosed with breast cancer	USA	RCT	2	112	8 weeks	Personal device
**Rossi 2010**	T1 DM	To evaluate whether a diabetes interactive diary could be effective in improving metabolic control in T1 DM	Italy<br>United Kingdom<br>Spain	RCT	2	130	6 months	Personal device
**Rossi 2013**	T1 DM	To evaluate the efficacy of a diabetes interactive diary on metabolic control and its impact on hypoglycemic episodes	Italy	RCT	2	127	6 months	Personal device
**Ryan 2012**	Asthma	To determine whether mobile phone-based monitoring improves asthma control and patient self-efficacy compared with standard paper-based monitoring	UK	RCT	2	278	6 months	Personal device

Author year	Target condition	Study aims	Country of study	Design	Study arms	Sample size	Follow-up interval	Device strategy
**Santo 2018**	CHD	To evaluate the effectiveness and feasibility of using publicly available high-quality medication reminder apps to improve medication adherence in patients with CHD	Australia	RCT	3	166	3 months	Personal device
**Seto 2012**	Heart failure	To assess the effects of a highly automated and user-centered mobile phone-based telemonitoring system on self-care and clinical management	Canada	RCT	2	100	6 months	Study device
**Shin 2016**	Chronic stroke	To investigate the preliminary efficacy of a smartphone-based visual feedback trunk control training in stroke patients	South Korea	RCT	2	24	4 weeks	Study device
**Skrovseth 2015**	T1 DM	To investigate whether a data-driven feedback module through a mobile app can improve self-management for T1 DM	Norway	RCT	2	30	8 weeks (Group 1)<br><br>10 weeks (Group 2)	Personal device
**Stukus 2018**	Asthma	To verify if children with a history of persistent asthma would benefit from the use of an app for a 6-month period in terms of decreased asthma-related ED visits and/or hospitalizations	USA	RCT	2	200	6 months	Personal device
**Sun 2017**	Various cancer types	To test the feasibility of Intelligent Pain Management System, the usage satisfaction, and QoL in cancer patients	China	RCT	2	46	14 days	Study device
**Sun 2019**	T2 DM	To determine whether a diabetes management system based on mobile phones is suitable for older patients	China	RCT	2	91	6 months	Personal device
**Tabak 2013**	COPD	To examine the effect of a telerehabilitation intervention compared with usual care in patients with COPD	The Netherlands	Pilot RCT	2	34	1 month	Study device
**Torbjorsen 2014**	T2 DM	To assess whether the use of an app, with and without a theory-based health counseling intervention, was superior to usual care in terms of glycemic control	Norway	RCT	3	151	12 months	Study device
**Varnfield 2014**	Post-myocardial infarction	To test whether a smartphone-based home care model is effective in improving cardiac rehabilitation use in post-MI patients	Australia	RCT	2	120	6 months	Study device
**Vorrink 2016**	COPD	To test whether a mHealth intervention, started after discharge from a 12-week pulmonary rehabilitation programme can enhance or maintain physical activity compared to usual care in patients with COPD	The Netherlands	RCT	2	157	6 months	Study device
**Vuorinen 2014**	Heart failure	To investigate if the multidisciplinary care of heart failure patients could be improved with telemonitoring	Finland	RCT	2	94	6 months	Study device
**Wang 2014**	COPD	To investigate whether home-based exercise training program can reduce inflammatory biomarkers in patients with COPD	Taiwan	Pilot RCT	2	30	6 months	Study device
**Wang 2018**	T2 DM	To assess the feasibility of a mHealth-enhanced intervention and compare its preliminary efficacy in improving glycemic outcomes among overweight or obese adults with T2DM	USA	Pilot RCT	2	26	6 months	Study device
**Wang 2019**	T2 DM	To explore the clinical effect of continuous care for patients with T2 DM using a mobile health app by comparing it with traditional discharge nursing	China	RCT	2	120	6 months	Personal device

Author year	Target condition	Study aims	Country of study	Design	Study arms	Sample size	Follow-up interval	Device strategy
**Wayne 2015**	T2 DM	To test the effectiveness of a mobile phone-based health coaching protocol in reducing the HbA1c of patients with T2 <DM from a lower-SES community	Canada	RCT	2	131	6 months	Study device
**Yang 2019**	Various cancer types	To develop and test an app for pain management among Chinese cancer patients discharged from hospital treatment	China	Pilot RCT	2	58	4 weeks	Study device
**Zairina 2016**	Asthma	To evaluate the efficacy of a telehealth programme in improving asthma control during pregnancy	Australia	RCT	2	72	6 months	Study device
**Zhou 2016**	T1 DM<br>T2 DM	To estimate the impact of the app on glycated hemoglobin	China	Pilot RCT	2	100	3 months	Personal device
**Zhu 2018**	Breast cancer	To test if participants receiving an e-support program would show significant better health outcomes compared with care as usual participants	China	RCT	2	114	12 weeks	Personal device

Abbreviations: BCT (behavior change technique); CHD (coronary heart disease); COPD (chronic obstructive pulmonary disease); CVD (cardiovascular disease); DM (diabetes mellitus); DMO (digital medicine offering); ED (emergency department); MI (myocardial infarction); NSCLC (non-small cell lung cancer); PA (physical activity); QoL (quality of life); RCT (Randomized Controlled Trial); SES (socio-economic status); T1 DM (type 1 diabetes mellitus); T2 DM (type 2 diabetes mellitus).

Supplementary Table 2 – App design and development factors considered by the included studies (n = 69)

Author year	Supporting behavioral theory (name)	User involvement in design	Healthcare professional involvement in design	Data security and privacy considerations	Pilot testing
**Agarwal 2019**	Yes (Transtheoretical Model of Behavior Change)	No	No	Yes	Yes
**Alanzi 2018**	Yes (The integrated theory of health behavior change)	No	No	No	No
**Athilingam 2017**	Yes (Learning theories including Mayer's Cognitive Theory of Multimedia Learning, Sweller's Cognitive Load, Instructional Design Approach, and Problem-Based Learning)	Yes	Yes	Yes	Yes
**Baron 2017**	Yes (Bandura's social cognitive theory, Leventhal's model of illness beliefs, and Davis's Technology Acceptance Model)	Yes	Yes	No	No
**Bee 2016**	No	No	No	No	Pilot study
**Boer 2019**	No	Yes	Yes	Yes	Yes
**Burckhardt 2018**	No	No	No	No	No
**Castensoe 2018**	No	Yes	Yes	Yes	Yes
**Charpentier 2011**	No	No	No	Yes	Yes
**Cingi 2015**	No	No	No	No	No
**Dang 2017**	No	No	No	Yes	No
**Drion 2015**	No	No	No	No	No
**Egbring 2016**	No	No	No	Yes	No
**Eyles 2017**	No	No	No	No	No
**Farmer 2017**	No	Yes	Yes	Yes	Yes
**Foley 2016**	No	No	No	Yes	Pilot study
**Forjouh 2014**	No	No	No	No	Yes
**Franc 2019**	No	No	No	Yes	Yes
**Frias 2017**	No	No	No	No	No
**Goyal 2018**	No	Yes	Yes	Yes	Yes
**Grady 2017**	No	No	No	No	Yes
**Greer 2019**	Yes (Cognitive Behavioral Therapy)	No	Yes	No	No
**Hagglund 2015**	No	Yes	Yes	No	No
**Hochsmann 2019**	Yes (Self-determination theory)	Yes	No	Yes	No
**Huang 2019**	No	No	No	Yes	Pilot study

Author year	Supporting behavioral theory (name)	User involvement in design	Healthcare professional involvement in design	Data security and privacy considerations	Pilot testing
**Ji 2019**	No	Yes	No	No	Yes
**Johnston 2016**	No	No	Yes	No	No
**Karhula 2015**	No	No	No	No	No
**Kearney 2009**	No	Yes	No	Yes	Yes
**Kim 2016**	No	No	No	No	No
**Kirwan 2013**	No	No	No	No	No
**Klee 2018**	No	Yes	Yes	Yes	No
**Kleinman 2017**	Yes (health belief model, health action process approach, theory of planned behavior, self-efficacy)	No	No	No	No
**Kosse 2019**	Yes (Common Sense Model of Self-Regulation)	Yes	Yes	Yes	No
**Kwon 2018**	No	Yes	Yes	Yes	Yes
**Labovitz 2017**	No	No	No	Yes	No
**Liu 2016**	No	No	No	Yes	No
**Logan 2012**	No	Yes	Yes	Yes	Yes
**Lyu 2016**	No	No	No	No	No
**Moore 2014**	No	No	No	No	No
**Morawski 2018**	No	No	No	No	Yes
**Or 2016**	No	No	No	No	Pilot study
**Orsama 2013**	Yes (Information-Motivation-Behavioral Skills Model)	No	No	No	No
**Quinn 2008**	No	No	Yes	Yes	Pilot study
**Quinn 2011**	No	No	Yes	Yes	Yes
**Rosen 2018**	No	No	No	Yes	Yes
**Rossi 2010**	No	No	No	No	Yes
**Rossi 2013**	No	No	No	No	Yes
**Ryan 2012**	No	Yes	No	Yes	Yes
**Santo 2018**	No	No	No	No	Yes
**Seto 2012**	No	Yes	Yes	Yes	Yes
**Shin 2016**	No	No	No	No	No
**Skrovseth 2015**	No	Yes	No	No	No
**Stukus 2018**	No	No	No	Yes	Yes
**Sun 2017**	No	No	No	No	Yes
**Sun 2019**	No	No	No	No	No

Author year	Supporting behavioral theory (name)	User involvement in design	Healthcare professional involvement in design	Data security and privacy considerations	Pilot testing
**Tabak 2013**	No	No	No	No	No
**Torbjorsen 2014**	No	Yes	No	Yes	Yes
**Varnfield 2014**	No	No	No	No	No
**Vorrink 2016**	No	Yes	No	Yes	Yes
**Vuorinen 2014**	No	No	No	Yes	No
**Wang 2014**	No	No	No	No	Pilot study
**Wang 2018**	Yes (Self-regulation and social learning theory)	No	No	No	Yes
**Wang 2019**	No	No	No	No	No
**Wayne 2015**	No	Yes	No	Yes	Yes
**Yang 2019**	No	No	Yes	No	Pilot study
**Zairina 2016**	No	No	No	Yes	No
**Zhou 2016**	No	No	No	Yes	Pilot study
**Zhu 2018**	Yes (Bandura' self-efficacy theory and social exchange theory)	Yes	Yes	Yes	No

Supplementary Table 3 – Features of the mobile app interventions (n = 69)

Author year	Technology automation *Level*	Technology automation *Notes*	Additional professional involvement	Professionals involved	Type of assistance	Corresponding clinician app
**Agarwal 2019**	High	Customized, evidence-based messages	No	-	-	No
**Alanzi 2018**	Low	App goal is to increase disease awareness	No	-	-	No
**Athilingam 2017**	High	Self-care intervention with algorithm that classifies patients into 4 zones	No	-	-	No
**Baron 2017**	Low	Review by MTH nurse that takes action based on the recorded inputs	Yes	MTH nurse	Access to data, feedback on OOR clinical readings, lifestyle education, insulin titration and referral to diabetes specialist nurse	No
**Bee 2016**	High	App suggests insulin dose, with maximum dose as a safety feature and general oversight by research staff	Yes	Research staff	General oversight and possibility to issue readings to endocrinologists	No
**Boer 2019**	High	Automated, tailored self-management advice based on a Bayesian prediction model	No	-	-	No
**Burckhardt 2018**	Low	Remote monitoring with information shared via cloud with up to five individuals	No	-	-	No
**Castensoe 2018**	Low	No automatic feedback on data	Yes	Healthcare Providers	Message exchange with participants	No
**Charpentier 2011**	High	Automatic algorithm for the adjustment of carbohydrate ratio	Yes	Medical Staff	Teleconsultations	No
**Cingi 2015**	Low	Treatment data are shared with physicians that can address urgent messages	Yes	Physicians	Receipt of urgent messages which triggered a voice notification, with the possibility to send 140-character messages and informational nudges	Yes
**Dang 2017**	Low	Data collection that triggered study coordinator intervention	Yes	Study coordinator	Contact whenever there were responses suggestive of deterioration of HF	No
**Drion 2015**	Low	App works as an ediary where parameters are reported	No	-	-	No
**Egbring 2016**	Low	Mobile app mostly acts as an ediary	Yes	Physicians	Review of input data during scheduled visits	No
**Eyles 2017**	Low	App only provides information	No	-	-	No
**Farmer 2017**	High	Software generated safety alerts that had to be addressed by clinicians	Yes	Respiratory clinicians	Contact via telephone or message if clinically important data were detected	No

Author year	Technology automation *Level*	Technology automation *Notes*	Additional professional involvement	Professionals involved	Type of assistance	Corresponding clinician app
**Foley 2016**	Low	App aimed at sharing information	No	-	-	No
**Forjouh 2014**	Low	Diabetes self-care software mostly works as an e-diary	Yes	Unclear	Classroom-based programmes for diabetes self-management	No
**Franc 2019**	High	Adaption of BI doses is automatic, but alert messages can be modified by the physicians	Yes	Physicians	Telephone consultation, despite the basal calculator required no prior validation by HCPs	No
**Frias 2017**	Low	Automatic medication adherence overview, with possibility to optimize target therapies by providers	Yes	Providers	Timely and targeted therapy optimization based on DMO data collected via a web portal	No (Provider web portal)
**Goyal 2018**	High	App includes Trend Wizard, an algorithm that detects OOR readings and identifies potential causes with no professional involvement	No	-	-	No
**Grady 2017**	Low	Participants received text-messages every 2 weeks from their HCP based on app data	Yes	Health Care Providers	Text-messages with diabetes related advice or specific adjustments and remote review of participants' SMBG levels	No (Website version of the app)
**Greer 2019**	Low	eTherapy simulation with no automation of technology in decision support	No	-	-	No
**Hagglund 2015**	High	Data collection was not shared with hospital, with self-care advices on weight management automatically provided by the app	No	-	-	No
**Hochsmann 2019**	High	An algorithm selects appropriate intensity progression for the exercise regimens	No	-	-	No
**Huang 2019**	High	Automatic medication adherence overview	No	-	-	No
**Ji 2019**	High	Exercise regimens automatically change based on achieved results	No	-	-	No (Web-portal)
**Johnston 2016**	High	Fully interactive patient support tool	No	-	-	No
**Karhula 2015**	Low	Data are transferred to the system, but are only used during calls with health coaches to discuss personal status	Yes	Health Coaches	Regular calls to provide assistance and support	No

Author year	Technology automation *Level*	Technology automation *Notes*	Additional professional involvement	Professionals involved	Type of assistance	Corresponding clinician app
**Kearney 2009**	High	App included self-care advice related to the symptoms reported, integrated with an evidence-based risk assessment tool that generated alerts to be addressed by clinicians	Yes	Clinicians	Evidence-based risk-assessment tool with amber alerts and red alerts (to be addressed within 1 hour of receipt)	No
**Kim 2016**	High	App gives daily signals about asthma control status, but also sends medical alerts to researchers that can make direct calls	Yes	Researchers	Medical alerts or emerging situations are also notified to researchers that can make direct calls to assist patient self-management	No
**Kirwan 2013**	Low	Data seen by CDEs that can send weekly personalized text-message feedback	Yes	Certified Diabetes Educator (CDE)	Weekly personalized text-message feedback on logs, diabetes questions, tips and reinforcement	No
**Klee 2018**	Low	Data are collected and diabetology team uses them to improve communication based on the input values	Yes	Diabetology team	Improved communications with feedback email based on the values input in the app	No
**Kleinman 2017**	High	Out-of-standard tests have automated questions to identify issues with coaches ultimately managing system-generated alerts	Yes	Health Coaches	Message exchange with patients and response to system-generated alerts	Yes
**Kosse 2019**	Low	Interactive intervention where pharmacists could monitor data and intervene	Yes	Pharmacists	Pharmacists can monitor scores, send additional movies, change settings and contact patients through chat function	No<br>(Desktop application)
**Kwon 2018**	High	Exercise regimens automatically change based on achieved results	No	-	-	No<br>(Website access)
**Labovitz 2017**	High	AI-based monitoring that confirms adherence and provides dosing instructions	No	-	-	No
**Liu 2016**	High	Patients receive management advice after uploading the diary data	No	-	-	No
**Logan 2012**	High	Automatic self-care messages after each reading, with critical alerts automatically sent by fax to clinicians	Yes	Clinicians	No additional clinic visits to physicians (automated self-visits), but clinicians receive critical alerts via fax	No
**Lyu 2016**	Low	Follow-up through WeChat app	Yes	Doctors	Doctor-led follow-up and consultations	Yes

Author year	Technology automation *Level*	Technology automation *Notes*	Additional professional involvement	Professionals involved	Type of assistance	Corresponding clinician app
**Moore 2014**	High	Personalized decision support encourages self-efficacy together with real-time messaging with nurse coaches	Yes	Nurse coaches	Real-time integrated messaging to support lifestyle change and medication adjustment	Yes (Nurse-coach tablet app)
**Morawski 2018**	High	Medication adherence stand-alone app	No	-	-	No
**Or 2016**	High	Automated feedback that supports self-management with minimized involvement of HCPs	No	-	-	No
**Orsama 2013**	High	Patients receive automated feedback messages linked to the reports of their health parameters, with nurses contacting patients if warranted	Yes	Nurses	Nurses contact patients if warranted, with high priority messages sent to personnel in charge of follow-up	No
**Quinn 2008**	High	Real-time feedback and suggested medication changes that the doctor has to decide to adopt	Yes	Health Care Providers	Clinicians decide whether to adopt recommendations and observe time-relevant trends	No
**Quinn 2011**	High	Patients receive automated, real-time messaging specific to the entered data	Yes	Diabetes educators	Educators can send electronic messages to the patient portal	No (Clinician portal)
**Rosen 2018**	Low	Meditation app with no need for patient directed feedbacks	No	-	-	No
**Rossi 2010**	High	Software automatically calculates the most appropriate insulin dose to be injected and fosters patient-physician communication based on collected data	Yes	Physicians or dietitians	Data collection and transmission of therapeutic and behavioral prescriptions	No
**Rossi 2013**	High	DID both calculates most appropriate insulin dose and facilitates regular physician feedback based on collected data	Yes	Physicians	Regular feedback based on the data collected by the DID	No
**Ryan 2012**	High	Patients are prompted to follow the agreed action plan, but incursion in red or amber zones triggered contact by asthma nurse	Yes	Asthma Nurse	Incursion in red or amber zones triggered contact by an asthma nurse	No
**Santo 2018**	High	Medication adherence stand-alone app	No	-	-	No

Author year	Technology automation *Level*	Technology automation *Notes*	Additional professional involvement	Professionals involved	Type of assistance	Corresponding clinician app
**Seto 2012**	High	Final messages or alerts sent to mobile phones, but cardiologists intervened when necessary	Yes	Cardiologists	Calls after receiving alerts or when contact was deemed warranted	No
**Shin 2016**	High	Automated feedback regarding trunk control during smartphone rehabilitation program	No	-	-	No
**Skrovseth 2015**	High	Data-driven feedback based on patients' data	No	-	-	No
**Stukus 2018**	High	Feedback based on electronic asthma treatment plan	No	-	-	No
**Sun 2017**	Low	Pain data collection that actives doctors' consultation	Yes	Doctors	Real-time consultation sessions on pain management with prompt advice	No
**Sun 2019**	Low	Continuous advice and dietary recommendations shared by professionals based on collected data	Yes	Medical Team and dietitians	Sharing of medical advice and dietary recommendations	No
**Tabak 2013**	High	Decision-support system-based advices to start medication with no professional involvement	No	-	-	No
**Torbjorsen 2014**	Low	App works as an electronic diabetes diary	Yes	Diabetes specialist nurses	Health counselling and responses to messages at least twice per week	No
**Varnfield 2014**	Low	Smartphone was preinstalled with a health diary, but updated data was used by mentors to provide personalized feedback during weekly consultations	Yes	Mentors	Teleconsultations to provide informed, personalized feedback on progress	No
**Vorrink 2016**	Low	Physiotherapists controlled PA data and could adjust goals (no automated adjustments)	Yes	Physiotherapists	Monitor PA data, adjust physical activity goals and send group or individual text messages	No (Web-portal)
**Vuorinen 2014**	High	Patients received automatic machine-based feedback, but nurses called patients if data differed markedly	Yes	Nurse	Contact patients if necessary based on data	No (Browsed-based interface)
**Wang 2014**	Low	Daily endurance exercise with no automated feedback	Yes	Healthcare professionals	Adherence reinforcement	No
**Wang 2018**	Low	Lifestyle changing intervention with no feedbacks provided	Yes	Unclear	11 group sessions at the community health centers (but no technology-related professional involvement)	No

**Author year**	**Technology automation *Level***	**Technology automation *Notes***	**Additional professional involvement**	**Professionals involved**	**Type of assistance**	**Corresponding clinician app**
**Wang 2019**	High	App automatically provides suggestions based on BG control, but physicians and diabetes nurse directly intervene for a list of activities	Yes	Physicians, Diabetes Specialist Nurses	Physicians: one-to-one interaction and overall health guide<br>Nurses: timely follow-up for one year	No
**Wayne 2015**	Low	Health coaches monitored patient inputs and directed immediate attention to episodes of interest	Yes	Health Coaches	Control of participant inputs and attention to episodes of progress, relapse or resistance	No
**Yang 2019**	High	System prompts patients with a medication order to follow based on data input, but real-time consultations with cancer team were available	Yes	Cancer Pain Management Team	Real-time consultation to support patients	No
**Zairina 2016**	High	Automated feedback message was sent weekly based on national guidelines, but HCPs would intervene if additional unscheduled changes were needed	Yes	Healthcare professionals	Intervene if any medication changes or asthma-related unscheduled visits were needed	No
**Zhou 2016**	Low	Advice is directly received from the study team	Yes	Clinicians	Advice and feedback for patients	Yes
**Zhu 2018**	Low	Forum with questions and information exchange	Yes	Moderators (Health Care Professional)	Moderators read all messages every day and provide expert advice to women's questions	No

Abbreviations: AI (artificial intelligence); BG (blood glucose); BI (basal insulin); CDE (certified diabetes educator); DID (diabetes interactive diary); DMO (digital medicine offering); HCP (healthcare professional); MTH (mobile telehealth); OOR (out of range); PA (physical activity); SMBG (self-monitoring blood glucose).

Supplementary Table 4 – Effect of studies (n=56)

Author year	Outcome measure	Core Area	Outcome domain	Results
**Agarwal 2019**	HbA1c	II. Physiological/clinical outcomes	5. Endocrine outcomes	Neutral
**Alanzi 2018**	Diabetes knowledge (DKT)	III. Life impact	29. Cognitive functioning	Positive
**Baron 2017**	HbA1c	II. Physiological/clinical outcomes	5. Endocrine outcomes	Neutral
**Boer 2019**	Exacerbation-free weeks	II. Physiological/clinical outcomes	22. Respiratory, thoracic and mediastinal	Neutral
**Burckhardt 2018**	Parental fear of hypoglycemia	IV. Resource use	37. Societal/carer burden	Positive
**Castensoe 2018**	HbA1c	II. Physiological/clinical outcomes	5. Endocrine outcomes	Positive
**Charpentier 2011**	HbA1c	II. Physiological/clinical outcomes	5. Endocrine outcomes	Positive
**Cingi 2015**	Quality of life (ACT)	III. Life impact	30. Global quality of life	Positive
**Dang 2017**	Self-efficacy (SECD)	III. Life impact	28. Emotional functioning / Wellbeing	Positive
**Drion 2015**	Quality of life (SF-36)	III. Life impact	30. Global quality of life	Neutral
**Egbring 2016**	Adverse events	V. Adverse events	38. Adverse events/effects	Neutral
**Eyles 2017**	Salt content of food purchases	II. Physiological/clinical outcomes	14. Metabolism and nutrition	Positive
**Farmer 2017**	COPD-specific health status	II. Physiological/clinical outcomes	22. Respiratory, thoracic and mediastinal	Neutral
**Forjuoh 2014**	HbA1c	II. Physiological/clinical outcomes	5. Endocrine outcomes	Neutral
**Franc 2019**	HbA1c	II. Physiological/clinical outcomes	5. Endocrine outcomes	Positive
**Frias 2017**	SBP	II. Physiological/clinical outcomes	3. Cardiac outcomes	Positive
**Goyal 2018**	HbA1c	II. Physiological/clinical outcomes	5. Endocrine outcomes	Neutral
**Grady 2017**	HbA1c	II. Physiological/clinical outcomes	5. Endocrine outcomes	Neutral
**Greer 2019**	Anxiety Rating Scale (HAM-A)	III. Life impact	28. Emotional functioning / Wellbeing	Neutral
**Hagglund 2015**	Disease-specific self-care (EHFScB-9)	III. Life impact	25. Physical functioning	Positive
**Hochsmann 2019**	Daily physical activity	III. Life impact	25. Physical functioning	Positive
**Huang 2019**	Self-reported barriers to medication adherence (ASK-12)	III. Life impact	32. Delivery of care	Positive
**Ji 2019**	Pulmonary function parameters (6MWD)	III. Life impact	25. Physical functioning	Neutral
**Johnston 2016**	Adherence to ticagrelor	III. Life impact	32. Delivery of care	Positive
**Karhula 2015**	Quality of life (SF-36)	III. Life impact	30. Global quality of life	Neutral
**Kirwan 2013**	HbA1c	II. Physiological/clinical outcomes	5. Endocrine outcomes	Positive
**Klee 2018**	HbA1c	II. Physiological/clinical outcomes	5. Endocrine outcomes	Neutral
**Kleinman 2017**	HbA1c	II. Physiological/clinical outcomes	5. Endocrine outcomes	Positive
**Kosse 2019**	Self-reported medication adherence (MARS)	III. Life impact	32. Delivery of care	Neutral
**Kwon 2018**	Change in respiratory function parameters (6MWT)	III. Life impact	25. Physical functioning	Neutral
**Labovitz 2017**	Medication adherence (Pill count)	III. Life impact	32. Delivery of care	Neutral
**Logan 2012**	SBP	II. Physiological/clinical outcomes	3. Cardiac outcomes	Positive
**Lyu 2016**	Quality of life (EORTC QLQ-H&N35)	III. Life impact	30. Global quality of life	Neutral

Author year	Outcome measure	Core Area	Outcome Domain	Results
**Moore 2014**	SBP / DBP	II. Physiological/clinical outcomes	3. Cardiac outcomes	Positive
**Morawski 2018**	Self-reported medication adherence (MARS)	III. Life impact	32. Delivery of care	Positive
**Orsama 2013**	HbA1c	II. Physiological/clinical outcomes	5. Endocrine outcomes	Neutral
**Quinn 2011**	HbA1c	II. Physiological/clinical outcomes	5. Endocrine outcomes	Positive
**Rosen 2018**	Quality of life (FACT-B)	III. Life impact	30. Global quality of life	Positive
**Rossi 2010**	HbA1c	II. Physiological/clinical outcomes	5. Endocrine outcomes	Neutral
**Rossi 2013**	HbA1c	II. Physiological/clinical outcomes	5. Endocrine outcomes	Neutral
**Ryan 2012**	Asthma control (ACQ)	II. Physiological/clinical outcomes	22. Respiratory, thoracic and mediastinal	Neutral
**Santo 2018**	Self-reported medication adherence (MARS)	III. Life impact	32. Delivery of care	Positive
**Seto 2012**	Self-care (SCHFI)	III. Life impact	25. Physical functioning	Neutral
**Skrovseth 2015**	OOR events	II. Physiological/clinical outcomes	5. Endocrine outcomes	Neutral
**Stukus 2018**	Asthma-related visits and hospitalizations	IV. Resource use	34. Economic	Neutral
**Tabak 2013**	Activity level (Steps per day)	III. Life impact	25. Physical functioning	Neutral
**Torbjorsen 2014**	HbA1c	II. Physiological/clinical outcomes	5. Endocrine outcomes	Neutral
**Varnfield 2014**	Uptake of CR programme	III. Life impact	32. Delivery of care	Positive
**Vorrink 2016**	Physical activity (steps/day)	III. Life impact	25. Physical functioning	Neutral
**Vuorinen 2014**	HF-related hospital days	IV. Resource use	35. Hospital	Neutral
**Wang 2018**	HbA1c	II. Physiological/clinical outcomes	5. Endocrine outcomes	Neutral
**Wayne 2015**	HbA1c	II. Physiological/clinical outcomes	5. Endocrine outcomes	Neutral
**Yang 2019**	Pain Management	II. Physiological/clinical outcomes	9. General outcomes	Positive
**Zairina 2016**	Asthma Control (ACQ-7)	II. Physiological/clinical outcomes	22. Respiratory, thoracic and mediastinal	Neutral
**Zhou 2016**	HbA1c	II. Physiological/clinical outcomes	5. Endocrine outcomes	Positive
**Zhu 2018**	Self-efficacy (SICPA)	III. Life impact	28. Emotional functioning / Wellbeing	Positive

Abbreviations: 6MWD (6-minute Walk Distance); 6MWT (6-minute Walking Test); ACQ (Asthma Control Questionnaire); ACT (Asthma Control Test); ASK-12 (Adherence Starts with Knowledge 12); CR (Cardiac Rehabilitation); DBP (Diastolic Blood Pressure); DKT (Diabetes Knowledge Test); EHFScB-9 (European Heart Failure Self-care Behaviour scale revised); EORTC QLQ-H&N35 (European Organisation for Research and Treatment of Cancer Quality of Life Questionnaire Head and Neck Module); FACT-B (Functional Assessment of Cancer Therapy – Breast); HAM-A (Hamilton Anxiety Rating Scale); HF (Heart Failure); MARS (Medication Adherence Rating Scale); OOR (Out-of-Range); SBP (Systolic Blood Pressure); SECD (Self-efficacy for managing chronic disease); SF-36 (Short form health survey 36); SFCHI (Self-Care of Heart Failure Index); SICPA (Stanford Inventory of Cancer Patient Adjustment).

Supplementary Table 5 – Inclusion and exclusion criteria for study eligibility.

**Inclusion criteria**	Prospective studies with a randomized design (i.e., parallel-group trials, cluster-randomized trials, cross-over trials, as well as other recent study designs specifically developed for digital technologies)
	Participants of any age with one of the four main noncommunicable diseases (i.e., cardiovascular diseases, cancers, chronic respiratory diseases, and diabetes)
	An intervention group which received an app-based intervention delivered through a smartphone or a tablet
	A comparator group which received either usual care or other, lower-intensity digital interventions, such as telephone follow-up, text messaging or a simplified version of the app
	Any type of quantitatively measured outcome reported for both the intervention and the comparator group
	Peer-reviewed articles published in English after 2008
**Exclusion criteria**	App intervention only available to healthcare professionals, with no related app version for patients
	Studies focusing on feasibility and usability only, with no attempt to evaluate, even preliminarily, the effectiveness of the intervention
	Mobile interventions relying solely on phone calls, web-based servers or SMS technology
	Multifactorial interventions in which the app was just one component and it was not possible to isolate its contribution
	Studies non-comparative in design
	A study population of healthy or at-risk individuals
	Reviews, study protocols, letters to the editor, conference proceedings and editorials

***Supplementary Note 1** – Literature search strategy for Medline (OVID interface)*

1. (mobile or mHealth or cellular phone* or app* or iphone* or phone* or smartphone*).mp.
2. exp Cell Phones/
3. exp Smartphone/
4. exp Mobile Applications/
5. 1 OR 2 OR 3 OR 4
6. (chronic disease* or CVD or CHD or coronary heart disease* or cardiovascular disease* or stroke* or myocardial infarction or cancer or tumo*r or chronic respiratory disease* or asthma or COPD or diabet* or T2DM or T1DM or IDDM or NIDDM or DM or T1D or T2D or MODY).mp.
7. 5 AND 6
